# Lessons from a COVID-19 hospital, Republic of Korea

**DOI:** 10.2471/BLT.20.261016

**Published:** 2020-10-06

**Authors:** Mhinjine Kim, Ji Yeon Lee, Jae Seok Park, Hyun Ah Kim, Miri Hyun, Young-Sung Suh, Sung Il Nam, Woo Jin Chung, Chi-Heum Cho

**Affiliations:** a School of Public Health, University of Illinois at Chicago, Chicago, United States of America.; b Department of Internal Medicine, Keimyung University Dongsan Hospital, Daegu, Republic of Korea.; c Department of Family Medicine, Keimyung University Dongsan Hospital, Daegu, Republic of Korea.; d Department of Otorhinolaryngology, Keimyung University Dongsan Hospital, Daegu, Republic of Korea.; e Department of Gastroenterology, Keimyung University Dongsan Hospital, Daegu, Republic of Korea.; f Department of Obstetrics and Gynaecology, Keimyung University Dongsan Hospital, 1035, Dalgubeol-daero, Dalseo-gu, Daegu, 42601, Republic of Korea.; Correspondence to Chi-Heum Cho (email: c0035@dsmc.or.kr).

## Abstract

**Objective:**

To document the experiences of converting a general hospital to a coronavirus disease 2019 (COVID-19) designated hospital during an outbreak in Daegu, Republic of Korea.

**Methods:**

The hospital management formed an emergency task force team, whose role was to organize the COVID-19 hospital. The task force used different collaborative channels to redistribute resources and expertise to the hospital. Leading doctors from the departments of infectious diseases, critical care and pulmonology developed standardized guidelines for treatment coherence. Nurses from the infection control team provided regular training on donning and doffing of personal protective equipment and basic safety measures.

**Findings:**

Keimyung University Daegu Dongsan hospital became a red zone hospital for COVID-19 patients on 21 February 2020. As of 29 June 2020, 1048 COVID-19 patients had been admitted to the hospital, of which 22 patients died and five patients were still being treated in the recovery ward. A total of 906 health-care personnel worked in the designated hospital, of whom 402 were regular hospital staff and 504 were dispatched health-care workers. Of these health-care workers, only one dispatched nurse acquired COVID-19. On June 15, the hospital management and Daegu city government decided to reconvert the main building to a general hospital for non-COVID-19 patients, while keeping the additional negative pressure rooms available, in case of resurgence of the disease.

**Conclusion:**

Centralized coordination in frontline hospital operation, staff management, and patient treatment and placement allowed for successful pooling and utilization of medical resources and manpower during the COVID-19 outbreak.

## Résumé

**Objectif:**

Documenter la transformation d'un hôpital général en hôpital de référence pour la maladie à coronavirus 2019 (COVID-19) durant une épidémie à Daegu, en République de Corée.

**Méthodes:**

La direction de l'hôpital a constitué une équipe opérationnelle d'urgence chargée de l'organisation au sein de l'hôpital COVID-19. Cette équipe a employé différents moyens de collaboration pour rediriger les ressources et l'expertise vers l'hôpital. Les médecins à la tête des départements des maladies infectieuses, des soins intensifs et de pneumologie ont formulé des directives standardisées afin d'assurer un traitement cohérent. Les infirmières affectées au contrôle des infections ont régulièrement dispensé des formations consacrées au port et au retrait des équipements de protection personnelle, ainsi qu'aux mesures de sécurité élémentaires.

**Résultats:**

Le 21 février 2020, l'hôpital Keimyung University Daegu Dongsan est devenu un établissement de zone rouge pour les patients atteints de la COVID-19. Entre cette date et le 29 juin 2020, il a admis 1048 patients COVID-19 et déploré 22 décès; cinq patients sont toujours hospitalisés en soins intensifs. Au total, 906 professionnels de la santé ont travaillé dans cet hôpital de référence: 402 faisaient déjà partie du personnel de l'établissement et 504 y ont été dépêchés. Sur l'ensemble de ces soignants, seule une infirmière envoyée sur place a contracté la COVID-19. Le 15 juin, la direction de l'hôpital et les autorités municipales ont décidé de reconvertir le bâtiment principal en hôpital général afin d'accueillir les patients non atteints par la COVID-19, tout en gardant les chambres à pression négative supplémentaires pour faire face à une éventuelle recrudescence de l'épidémie.

**Conclusion:**

La coordination centralisée du fonctionnement de l'hôpital en première ligne, la gestion du personnel ainsi que le traitement et le placement des patients ont permis de regrouper et d'exploiter au mieux les ressources médicales et humaines durant l'épidémie de COVID-19.

## Resumen

**Objetivo:**

Registrar las experiencias de la conversión de un hospital general en un hospital designado para la enfermedad por coronavirus de 2019 (COVID-19) durante un brote epidémico en Daegu, República de Corea.

**Métodos:**

La dirección del hospital constituyó un equipo de trabajo de emergencia, cuya función era organizar el hospital COVID-19. El equipo de tareas utilizó diferentes medios de colaboración para redistribuir los recursos y los conocimientos técnicos al hospital. Los médicos más destacados de los servicios de enfermedades infecciosas, cuidados intensivos y neumología elaboraron unas directrices normalizadas para asegurar la uniformidad de los tratamientos. Los enfermeros del equipo de control de infecciones imparten capacitación de manera regular sobre cómo ponerse y quitarse el equipo de protección personal y sobre las medidas de seguridad básicas.

**Resultados:**

El hospital Keimyung University Daegu Dongsan se convirtió en un hospital de la zona roja para los pacientes con la COVID-19 el 21 de febrero de 2020. Al 29 de junio de 2020, 1048 pacientes con la COVID-19 fueron admitidos en el hospital, de los que 22 murieron y 5 fueron tratados en la planta de recuperación. 906 profesionales sanitarios trabajaban en el hospital designado, de los que 402 formaban parte del personal ordinario del hospital y 504 eran profesionales sanitarios temporales. De estos profesionales sanitarios, solo 1 enfermero temporal adquirió la COVID-19. El 15 de junio, la dirección del hospital y el gobierno municipal de Daegu decidieron reconvertir el edificio principal en un hospital general para los pacientes que no tenían la COVID-19, conservando las salas adicionales de presión negativa que se habían construido, en caso de que la enfermedad volviera a presentarse.

**Conclusión:**

La coordinación centralizada en el funcionamiento de la primera línea del hospital, la gestión del personal y el tratamiento y la ubicación de los pacientes permiten la agrupación y el uso satisfactorio de los recursos médicos y de la fuerza de trabajo durante el brote epidémico de la COVID-19.

## ملخص

الغرض توثيق تجارب تحويل مستشفى عام إلى مستشفى مخصص لعلاج مرض فيروس كورونا 2019 (كوفيد 19)، أثناء تفشي المرض في دايجو، بجمهورية كوريا.

الطريقة قامت إدارة المستشفى بتشكيل فريق عمل للطوارئ، كان دوره هو تنظيم مستشفى كوفيد 19. اعتمدت فرقة العمل قنوات تعاونية مختلفة لإعادة توزيع الموارد والخبرات على المستشفى. قام كبار الأطباء من أقسام الأمراض المعدية، والرعاية الحرجة، وأمراض الرئة، بوضع إرشادات موحدة لتحقيق الاتساق في العلاج. تقدم الممرضات من فريق مكافحة العدوى تدريبات منتظمة على ارتداء وخلع معدات الحماية الشخصية، وإجراءات السلامة الأساسية.

النتائج أصبح مستشفى دايجو دونجسان بجامعة كيم يونج، مستشفى بتصنيف المنطقة الحمراء لمرضى كوفيد 19، في 21 فبراير/شباط 2020. اعتبارًا من 29 يونيو/حزيران 2020، تم إدخال 1048 مريضًا مصابًا بكوفيد 19 إلى المستشفى، توفي منهم 22 مريضًا، وما زال خمسة مرضى قيد العلاج في وحدة النقاهة بالمستشفى. قام إجمالي 906 من العاملين في مجال الرعاية الصحية بالعمل في هذا المستشفى المخصص، من بينهم 402 من فريق عمل المستشفى المنتظمين، و504 من العاملين المنتدبين في مجال الرعاية الصحية. من بين هؤلاء العاملين في مجال الرعاية الصحية، أصيبت ممرضة منتدبة واحدة فقط بكوفيد 19. وفي 15 يونيو/حزيران، قررت كل من إدارة المستشفى وحكومة مدينة دايجو إعادة تحويل المبنى الرئيسي إلى مستشفى عام للمرضى غير المصابين بكوفيد 19، مع الحفاظ على غرف الضغط السلبي الإضافية المبنية، إذا ما عاود المرض الظهور.

الاستنتاج يسمح كل من التنسيق المركزي في تشغيل المستشفى في الخطوط الأمامية، وإدارة الموظفين، وعلاج المرضى وتوزيعهم، بالتجميع والاستخدام الناجح للموارد الطبية والقوى العاملة أثناء تفشي مرض كوفيد 19. ****

## 摘要

**目的:**

记录韩国大邱疫情爆发期间，将一家综合医院转变为新型冠状病毒肺炎指定救治医院的经验。

**方法:**

医院管理层组建了一支应急工作队，负责组织新型冠状病毒肺炎医院的工作。工作队通过不同的协作渠道向医院重新分配资源和专家经验。传染病科、重症监护科和肺病学科的权威医生制定了治疗一致性相关的标准化指南。感染控制小组的护士定期就个人防护设备的穿脱和基本安全措施提供培训。

**结果:**

2020 年 2 月 21 日，启明大学大邱东山医院成为面向新型冠状病毒肺炎患者的红区医院。截至 2020 年 6 月 29 日，共有 1048 名新型冠状病毒肺炎患者入院，其中 22 名患者死亡，另有 5 名患者仍在康复病房接受治疗。在该指定医院工作的医护人员共有 906 名，其中固定医院工作人员 402 人，派遣医护人员 504 人。在这些医护人员中，仅一名派遣护士感染了新型冠状病毒肺炎。6 月 15 日，医院管理层和大邱市政府决定将主楼改建为面向非新型冠状病毒肺炎患者的综合医院，同时保留新增的负压隔离病房，以防疾病复发。

**结论:**

通过集中协调前线医院运营、医护人员管理以及患者救治安置，能够在新型冠状病毒肺炎爆发期间有效地集中和利用医疗资源和人力。

## Резюме

**Цель:**

Задокументировать опыт преобразования больницы общего профиля в больницу, предназначенную для лечения коронавируса 2019 г. (COVID-19), во время вспышки в Тэгу, Республика Корея.

**Методы:**

Руководство больницы сформировало целевую группу оперативного реагирования, роль которой заключалась в организации больницы для лечения COVID-19. Целевая группа использовала различные каналы сотрудничества для передачи ресурсов и знаний в больницу. Ведущие врачи отделений инфекционных заболеваний, реанимации и пульмонологии разработали стандартизированные рекомендации для согласованности лечения. Медсестры из группы инфекционного контроля проводят постоянные тренинги по надеванию и снятию средств индивидуальной защиты, а также по основным мерам безопасности.

**Результаты:**

21 февраля 2020 года больница Тэгу Донгсан при Университете Кемён стала больницей красной зоны для пациентов с COVID-19. По состоянию на 29 июня 2020 года в больницу поступило 1048 пациентов с COVID-19, из которых 22 пациента умерли, а пять пациентов все еще проходят лечение в палатах для выздоравливающих. В указанной больнице работали в общей сложности 906 работников здравоохранения, из которых 402 были штатными сотрудниками больницы, а 504 — командированными работниками здравоохранения. Из этих работников здравоохранения только одна командированная медсестра заразилась COVID-19. 15 июня руководство больницы и городская администрация г. Тэгу решили снова переоборудовать главное здание в больницу общего профиля для пациентов, не инфицированных COVID-19, сохраняя построенные дополнительные палаты с отрицательным давлением на случай новой вспышки заболевания.

**Вывод:**

Централизованная координация действий по работе больницы первичного звена, управлению персоналом, лечению и размещению пациентов позволяет успешно объединять и использовать медицинские и трудовые ресурсы во время вспышки COVID-19.

## Introduction

The coronavirus disease 2019 (COVID-19) outbreak has affected almost all countries in the world. Such health crises call for hospitals and other health-care facilities to develop strategies to manage unprecedented numbers of patients while competing for a finite set of resources.^1^^–^^3^ Sharing countries’ experiences of handling the outbreak might translate into improved response to the outbreak. Although there are many research publications on clinical characteristics and treatment management of COVID-19,^4^^–^^6^ discussion on designated hospital operation and management remains limited. Therefore, we describe our experiences from a COVID-19 treatment hospital located in the city of Daegu, the COVID-19 epicentre in the Republic of Korea in the spring of 2020.

As of 29 June 2020, the country had reported 12 757 confirmed COVID-19 cases (including 1551 imported cases).^7^ Of these cases, 11 364 people have been discharged from isolation and 282 patients have died. Daegu has been most affected with 6906 confirmed cases (54.2%), followed by Gyeongsangbuk-do Province (1388 cases; 10.9%), the capital Seoul (1305 cases; 10.2%) and Gyeonggi-do Province (1200 cases; 9.4%).^8^

The first confirmed COVID-19 case was reported on 20 January 2020.^9^ Initial COVID-19 patients in the country were mainly visitors from China. On February 18, the first COVID-19 patient was confirmed in Daegu, a city with approximately 2.5 million inhabitants. Within a month, Daegu had over 6200 confirmed cases, many of which could be linked to the Shincheonji Church. On 15 March, the government designated Daegu as one of four special disaster zones heavily affected by COVID-19.^10^ This announcement was the first time the government declared a special disaster zone due to the disproportionate impact of an infectious disease on its population.

To respond to the outbreak and improve patient care, Daegu city government and the hospital management agreed to convert Keimyung University Daegu Dongsan Hospital to a COVID-19 designated hospital. Here we describe our experiences in anticipating, absorbing and adapting to an increase in the surge of patients during the COVID-19 outbreak.

## Methods

### Local setting

The private Keimyung University Dongsan Medical Center has three hospitals, of which two are located in Daegu: the tertiary hospital Keimyung University Dongsan Hospital with 992 beds and secondary level Daegu Dongsan Hospital with almost 1000 beds capacity, but only 216 beds in use before the COVID-19 outbreak.^11^ When the Keimyung University Dongsan Hospital was transferred to a newly built hospital in the western area of Daegu in April 2019, the old hospital building in the centre of the city became Daegu Dongsan Hospital. The two hospitals are 10km apart, a journey of about 20–30 mins by car. In January 2020, the daily number of outpatients was approximately 3300 in Daegu Dongsan Hospital and 565 in Keimyung University Dongsan Hospital (Keimyung University Dongsan Medical Center, Daegu, Republic of Korea, unpublished data, August 2020).

At the beginning of the outbreak, the policy in the country was to hospitalize all confirmed COVID-19 cases. However, on 21 February 2020, the accumulated number of patients in Daegu was 59, yet only 15 patients were able to be hospitalized due to the limited number of negative pressure rooms in the hospitals. To ensure that all COVID-19 patients could be admitted to a hospital, the Daegu city government agreed with the hospital management that the Daegu Dongsan Hospital should be the designated COVID-19 hospital in the city, since this hospital had a larger plot area and was easier to transform. The non-COVID-19 patients in Daegu Dongsan Hospital were therefore transferred to Keimyung University Dongsan Hospital.

### Approaches

#### Emergency task force team

The management of Daegu Dongsan Hospital focused all its resources and expertise needed for COVID-19 patient care. The two hospitals established a joint emergency task force team, which consisted of leading staff members in the divisions of medicine, infection control, nursing, laboratory medicine, radiology, administration, facilities, logistics, nutrition and public relations. To enable swift communication and decision-making, the emergency task force team resided in the designated hospital’s annex building. A daily face-to-face meeting allowed the head of each department to actively discuss different topics of patient care, staffing, medical supplies and issues that need to be resolved.

To reduce confusion in patient care, the heads of the department of infectious diseases, critical care and pulmonology had the ultimate authority. Doctors of different specialties participated in the COVID-19 patient care and they used group chats in the instant messaging application KakaoTalk for smartphones to discuss patient treatment in real time. The physician team, which included doctors from infectious disease, pulmonology, radiology and laboratory medicine, managed all areas of diagnosis and treatment of COVID-19 and infection control in the hospital. The task force assigned nurses to support the physician team. For example, the nurses managed the inpatient list and daily COVID-19 polymerase chain reaction (PCR) testing list, and reported the inpatient list and patients who had two consecutive negative PCR results to the government.

#### Converting the hospital

**Fig. 1:**
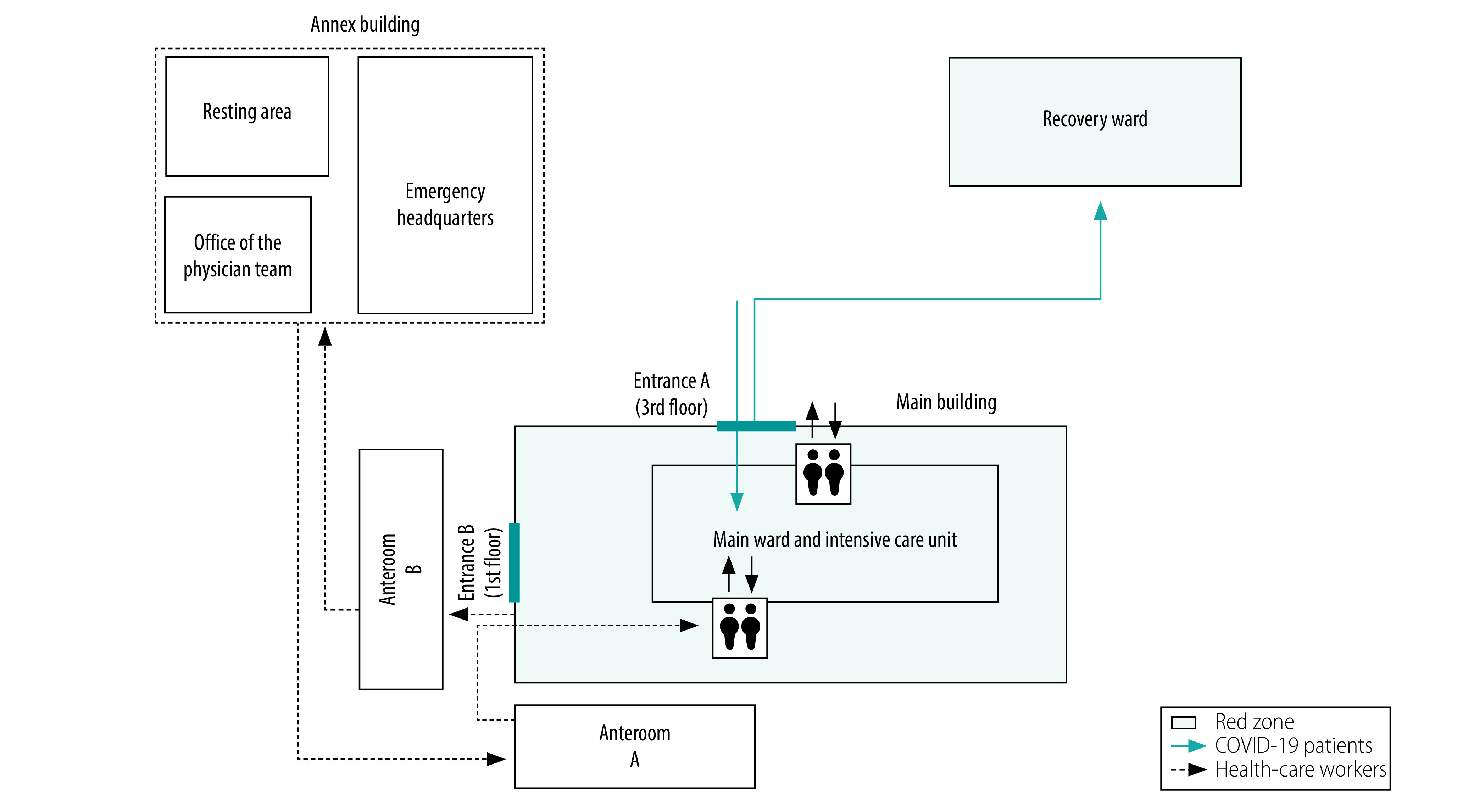
Schematic layout of a COVID-19 hospital in Daegu, Republic of Korea, 2020

Given the large number of patients but limited number of beds and negative pressure rooms, the task force decided to convert the entire main building of the hospital to a red zone on 21 February. With help from key health-care workers, primarily physicians of infectious diseases, who had experience in patient management and infection prevention and control of the Middle East respiratory syndrome, the hospital was transformed in one day. These key health-care workers managed the overall process to convert the main building to a red zone and suggested separated routes for medical staff and patients. Only employees wearing personal protective equipment could enter the main building through the entrance on the first floor, while COVID-19 patients entered the building through an entrance on the third floor. All other entrances were closed. Separate elevators were used for patients and staff, and line stickers were put on the floor to guide the route for patients and staff. Medical personnel could not enter the red zone without putting on personal protective equipment in Anteroom A (Fig. 1). 

COVID-19: coronavirus disease 2019.

Notes: Health-care workers put on personal protective equipment in anteroom A and removed it in anteroom B. Pathways of COVID-19 patients and health-care workers were separated in the main building.

All general wards, located on the fifth to eighth floor in the hospital, were now used for COVID-19 patients. Bed-ridden or elderly patients as well as patients with severe pneumonia were placed on the fifth floor so that they could be moved quickly if needed to the intensive care unit, also located on the fifth floor. At first, only three negative pressure rooms were available in the intensive care unit. We expanded the number of beds in the unit to 20 beds. At first, there was no time for creating individual negative pressure rooms in the intensive care unit, but we created a station surrounded by a dividing glass wall in the centre of the unit, allowing medical staff to monitor patients inside the station.

To minimize the time health-care workers spent in the hospital wards and to potentially reduce the infection risk, we placed smartphones in each ward. These devices were used for video consultations before ward rounds to check patients’ conditions.

To separate recovering patients from those who were more sick, the task force created a separate recovery ward in an additional hospital building (previously used as a research building).

Expensive and hard-to-obtain medical devices needed for the set-up of new intensive care units, such as extracorporeal membrane oxygenation and mechanical ventilators, were lent by other hospitals or were purchased with funding from nongovernmental organizations.

At the beginning of April, we constructed six additional negative pressure rooms in the general wards to expand the hospital’s capacity to isolate suspected COVID-19 patients who may require treatment. Before the construction, we transferred the COVID-19 patients on the relevant floor to other floors within the main building or to the recovery ward. After ventilating the area for 24 hours, we started the construction of the negative pressure rooms. The entire construction took 10 days and ended on 14 April 2020. Separate anterooms for donning and doffing of personal protective equipment were built, and interlocking doors and portable negative pressure equipment were installed.

#### Personal protective equipment

All health-care workers in contact with confirmed COVID-19 patients were instructed to wear coveralls, N95 mask, goggles or face shield and double gloves. When treating patients for more than 3–4 hours or in situations where aerosols could be generated (for example during intubation or suction), health-care workers wore a powered air purifying respirator. All medical personnel wore such respirators constantly while working in spaces with high probability of aerosol generation, such as in the intensive care units. Health-care workers discarded coveralls, N95 masks and gloves after use. Due to a shortage in supply of goggles and powered air purifying respirators, health-care workers reused them after the equipment had been sterilized. After the first use, powered air purifying respirators were first wiped with alcohol and then with a benzalkonium chloride tissue. After 1–2 weeks of use, we used ethylene oxide gas to sterilize them. We discarded reused powered air purifying respirators after they been sterilized with ethylene oxide gas once or twice, because safe performance of the respirator could not be guaranteed. We sterilized the goggles by cleaning them with 70% (vol./vol.) ethanol and wiping with dry tissues, and then disinfected them with ethylene oxide gas.

By having two separate anterooms (Fig. 1), one for donning and one for doffing, we aimed to avoid contamination of the donning area. In each anteroom, managers helped staff to put on and take off personal protective equipment and monitored the process. The anteroom also had video surveillance to ensure personal protective equipment was worn and removed in a safe manner.

All health-care workers received one KF94 or surgical mask per day to wear at all times when they resided in the clean zone.

#### Health workforce

We prioritized more experienced health-care workers and those with experience in other high-consequence pathogens to improve patient care and support less experienced health-care workers at the beginning of this outbreak.

In the early stages of the running of the COVID-19 hospital, the ordinary workforce was able to provide treatment to all COVID-19 patients. However, as the number of patients, beds and intensive care units increased, additional health-care workers were needed, not only to fill the gap in staffing but also to relieve fatigued personnel. Doctors and nurses from other parts of the country were rapidly dispatched to the hospital through different medical societies. First, public hospital doctors and nurses, military doctors, public health doctors and nurse officers were dispatched. Then, civilian nurses recruited from the health ministry and civilian doctors who were volunteering participated in COVID-19 patient treatment. The Medical Association, the Society of Critical Care Medicine and the Nurses Association of the Republic of Korea also helped to recruit volunteers. The national government covered most of housing fees, daily expenses, hazard pay and other costs associated with dispatchment of workforce. The medical and humanitarian assistance nongovernmental organization, Global Care, also partly supported the dispatchment.

To ensure safety of all health-care workers and to prevent nosocomial infection, nurses from the infection control team provided regular training on donning and doffing of personal protective equipment and basic safety measures, such as how to prevent virus exposure and what to do if one is exposed. The training sessions, which were given on-site at least twice every day, lasted about one hour. Before the hands-on training, participants were given instructions on wearing and removing of personal protective equipment. If the trainer deemed a participant to be unskilled, the participant was re-trained until a satisfactory level was reached. During training, we also provided N95 mask fit testing so individuals could select the most suitable N95 mask. We enabled regular check-ups of the masks by constructing an N95 mask fit test booth near Anteroom A where personal protective equipment was put on.

The emergency task force team instructed all health-care personnel not to eat face-to-face or communicate with one another during meals. In staff cafeteria, distances between all chairs were doubled and seats were rearranged so that staff members were not facing each other.

#### Development of local guidelines

To reduce confusion in patient treatment and negative outcomes, leading doctors from the departments of infectious diseases, critical care and pulmonology developed standardized guidelines for treatment coherence. They developed two treatment guidelines: one for patients with mild symptoms (i.e. mild illness, pneumonia without hypoxaemia) and one for patients who required critical care (i.e. severe pneumonia, acute respiratory distress syndrome, multiorgan failure, etc.). To ensure standardized care was given to all patients, the leading doctors announced the guidelines on the bulletin board and also shared in group chats in an instant messaging app. This approach reflects the recommendation^12^ that called for intensive care physicians to act as leaders to make sure standardized treatment is given to all patients with severe disease.^12^ For patients without pneumonia, younger than 60 years and with no comorbidities, health-care workers were only required to control their symptoms. Patients with pneumonia, chronic illnesses or who were older than 60 years received an antiviral agent (that is, hydroxychloroquine or ritonavir/lopinavir). For patients with rapid progression of hypoxia or for those in need of supplemental oxygen greater than 6 L/min, we administered intravenous steroid injection (methylprednisolone 30 mg).

To help with patient placement in the hospital, we developed a novel scoring system to predict progression to severe pneumonia in patients with COVID-19. The scoring system contained four independent predictive factors for progression (age, C-reactive protein, lactate dehydrogenase, haemoglobin) and each factor’s score was based on its regression coefficient, which we obtained through a multivariate logistic regression analysis. The risk score is the sum of the factor scores, and a patient’s risk score could range from 0 to 20 points (Box 1). We constructed two patient groups based on the risk scores: patients with low risk (0 to 8 points) and patients with high risk (9 to 20 points). Medical staff monitored high-risk patients more intensively than low-risk patients and placed high-risk patients in wards closer to the intensive care unit. Recently admitted, low-risk patients were placed in a separate ward.

Box 1 **Scoring system to identify COVID-19 patients with high risk of progression to severe pneumonia, Daegu, Republic of Korea, 2020**

**Age**

< 50 years: 0 points

50–59 years: 4 points

60–69 years: 5 points

70–79 years: 7 points

> 79 years: 10 points

**C-reactive protein**

< 1.4 mg/dL: 0 points

≥ 1.4 mg/dL: 3 points

**Lactate dehydrogenase**

< 500 U/L: 0 points

500–700 U/L: 2 points

> 700 U/L: 4 points

**Haemoglobin**

< 13.3 g/dL: 0 points

≥ 13.3 g/dL: 3 points

### Reconversion to general hospital

As the number of newly confirmed COVID-19 patients in Daegu declined and there were no more COVID-19 patients needing intensive care, the hospital management decided to reconvert the main building to a general hospital on 15 June 2020. 

To prepare for a new surge of COVID-19 cases, we maintained 154 beds in the recovery ward for COVID-19 patients presenting with mild symptoms. 

## Results

**Fig. 2:**
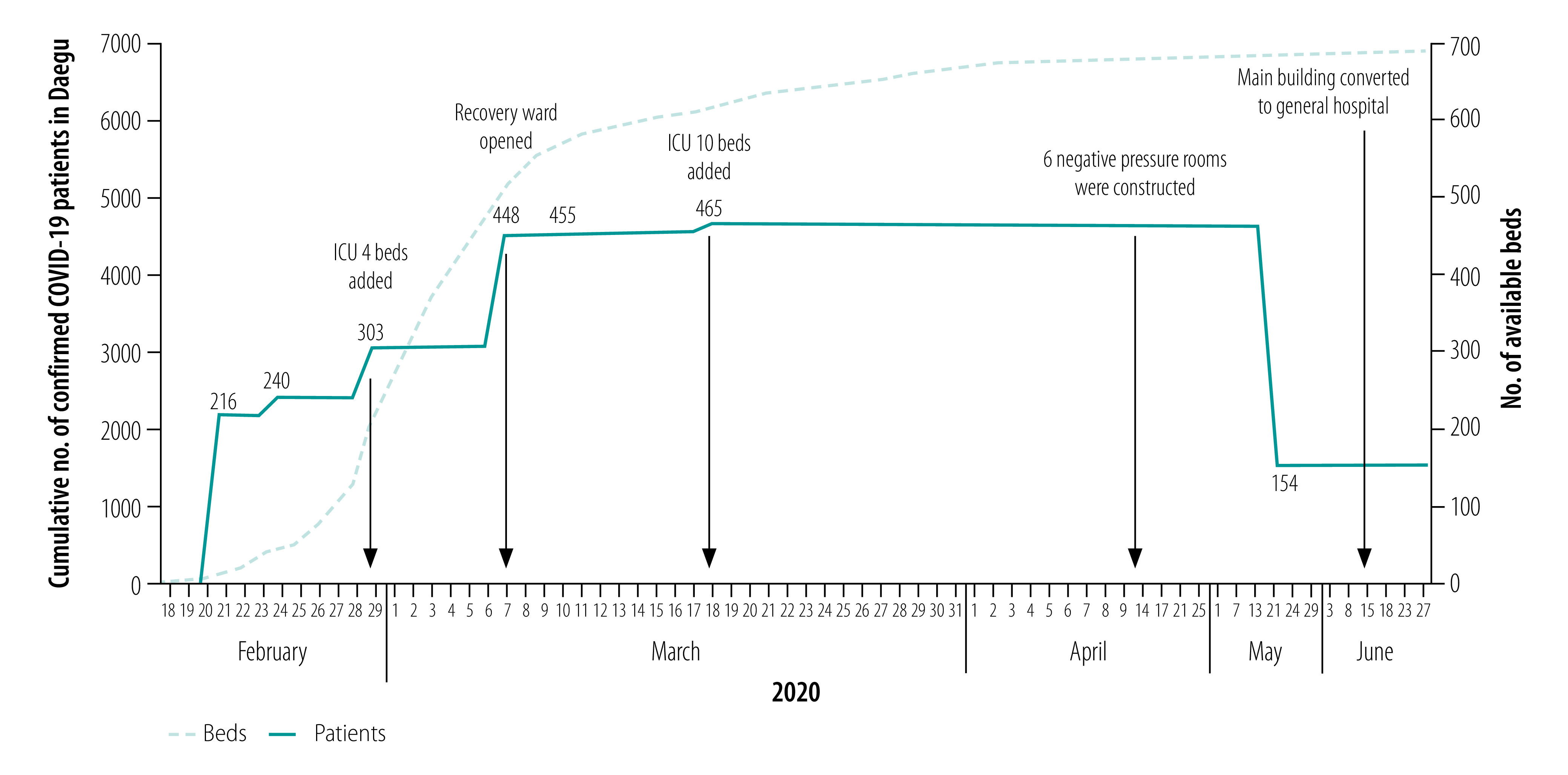
Number of beds in Keimyung University Daegu Dongsan Hospital and accumulated number of COVID-19 patients in Daegu, Republic of Korea, 18 February to 27 June 2020

COVID-19: coronavirus disease 2019; ICU: intensive care unit.

After the Daegu Dongsan Hospital was designated as a COVID-19 hospital, its total capacity increased from 216 beds and five wards, including one intensive care unit, to 465 beds and 10 wards, including two intensive care units (Fig. 2). As of 29 June 2020, a total of 1048 COVID-19 patients had been admitted to the hospital, which is the largest number of COVID-19 patients hospitalized in a single centre in the Republic of Korea. Out of the 1048 patients, 520 had pneumonia, 149 required oxygen therapy, 15 needed mechanical ventilation and three were on extracorporeal membrane oxygenation. Out of the 22 patients who died, 11 patients did not receive any intensive care due to do-not-resuscitate orders. As of 29 June, five patients were hospitalized in the recovery ward.

A total of 906 health-care personnel worked in the designated hospital, of whom 402 were regular hospital staff and 504 were dispatched health-care workers. Of these health-care workers, only one dispatched nurse acquired COVID-19.

On 21 May, the remaining COVID-19 patients hospitalized in the main building were transferred to the recovery ward. On June 15, the hospital management and Daegu city government decided to reconvert the main building to a general hospital for non-COVID-19 patients, while keeping the negative pressure rooms, three in the intensive care unit and six in the general ward.

## Discussion

Here we describe how a general hospital in the epicentre of a COVID-19 outbreak was transformed into a red zone hospital. Converting the entire hospital to a red zone ensured both isolation and care for COVID-19 patients as well as protection of health-care workers.

While city government and hospital management undertook many measures in response to the surge in cases, we believe three measures in particular played a pivotal role in controlling the outbreak and dealing with resource and personnel constraints. First, the decision to develop and operate a COVID-19-specialized red zone hospital was an emergency strategy that allowed efficient use of already limited personal protective equipment and medical personnel. In addition, the conversion enabled us to easily expand specific bed capacity. Second, involving experienced staff in setting up the emergency response system was key in providing well-orchestrated care provision and reducing avoidable work-related burden. Their leading roles and experience helped other staff members new to the infectious disease control field. Third, the coordinated approach taken by the government and the hospital allowed for pooling of much needed resources in the COVID-19 hospital and helped to preserve normal functions of other hospitals in the city. Patients in other hospitals diagnosed with COVID-19 were immediately transferred to the designated COVID-19 hospital. 

Despite the high number of health-care workers working at the hospital, only one acquired COVID-19. We believe that the thorough training on the use of personal protective equipment, the adequate supply of such equipment, the additional workforce and the social distancing rules for staff contributed to this positive outcome.

Making the hospital a COVID-19 designated hospital guaranteed an adequate supply of personal protective equipment, since the reconverted hospital received direct and prioritized assistance from the central government and Daegu city government. In addition, other entities, including the national Center for Disease Control and Prevention, companies and citizens, donated personal protective equipment to the hospital. Therefore, all health-care workers in contact with confirmed COVID-19 patients were able to wear personal protective equipment at all times. 

As the COVID-19 outbreak continued, we noted that health-care workers began to experience greater physical and mental fatigue. Therefore, placing empowered staff leaders at the designated hospital was important to boost morale and make workers feel valued, as well as protecting the health-care workforce. Another challenge was the non-medical personnel, such as cleaners and people distributing the meals, who were not familiar with infectious diseases and infection control. Without adequate training they posed a higher risk of virus exposure and nosocomial infection, which could lead to a serious personnel shortage. We therefore also provided training for them and gave them feedback.

We hope our experiences and lessons learnt while converting a hospital to a designated COVID-19 hospital will be useful for public health officials in other countries experiencing similar situations.
